# Non-metastatic castration-resistant prostate cancer in the epoch of PSMA-ligand PET/CT: twilight?

**DOI:** 10.1007/s12149-026-02195-z

**Published:** 2026-03-26

**Authors:** Pipitsa N. Valsamaki, Georgios Tsakaldimis

**Affiliations:** 1 Department of Nuclear Medicine, School of Medicine, Democritus University of Thrace, University Hospital of Alexandroupolis, Alexandroupolis, 68100 Greece; 2 Department of Urology, School of Medicine, Democritus University of Thrace, University Hospital of Alexandroupolis, Alexandroupolis, Greece

**Keywords:** PSMA-ligand PET/CT, Restaging, Non‑metastatic CRPC

## Abstract

The introduction of prostate-specific membrane antigen-ligand positron emission tomography/computerised tomography (PSMA-ligand PET/CT) into clinical practice has altered the prevalence of non‑metastatic (by conventional imaging) castration-resistant prostate cancer (nmCRPC). We herewith briefly review the biochemical and imaging parameters in nmCRPC and mainly focus on decoding disease localization using PSMA- ligand PET. The effective restaging with more accurate PSMA PET-based disease identification and characterization potentially signifies the entrance of the nmCRPC stage in the twilight zone. Moreover, it seems plausible that the small fraction of the nmCRPC invisible even on PSMA PET may constitute a latent phase of occult growth of the tumor cells. Supposedly, triggering of PSMA overexpression on the PCa cells under androgen deprivation therapy (ADT) through certain anticancer or other therapies such as protein kinase inhibitors (PKIs), dutasteride, or cholesterol-lowering regimes, might further hasten visualisation of micrometastases on PSMA-ligand PET, eventually transforming the nmCRPC stage into a defined, potentially curable target.

## Introduction

Among the established clinical indications of the novel prostate-specific membrane antigen-ligand positron emission tomography/computerised tomography (PSMA- ligand PET/CT), we hereby engage in decoding the localization of non‑metastatic (by conventional imaging) castration-resistant prostate cancer (nmCRPC). In patients with nmCRPC, the prostate-specific antigen (PSA) levels increase despite androgen deprivation therapy (ADT) without, however, having detectable local relapse or distant metastasis (Table 1) [1, 2]. New criteria were proposed to define the transformation of nmCRPC to metastatic disease by the expert committee of prostate cancer clinical investigators (the Prostate Cancer Clinical Trials Working Group 3, PCWG3). This transient stage of castration-resistant prostate cancer (nmCRPC - M0) roughly affects 10% of prostate cancer patients, whilst progression to radiologically overt metastatic disease occurs in up to 60% within 5 years [3]. The management of nmCRPC remains challenging for urologists and oncologists due to the clinical heterogeneity and the hazard of understaging the disease [4].

**Table 1:** Requisites defining nmCRPC

castration serum testosterone	< 50 ng/dL
Three consecutive rises in PSA	Two 50% increases above the nadir
PSA	> 2 ng/mL (EAU) or > 1 ng/mL (PCWG3)
Conventional imaging	Negative

nmCRPC: non-metastatic Castration-Resistant Prostate Cancer

PSA: Prostate-Specific Antigen

EAU: European Association of Urology, PCWG3: The Prostate Cancer Working Group 3

## Synopsis of biochemical and imaging biomarkers in nmCRPC

In 1981 PSA, a 33 kD glycoprotein, was identified almost exclusively in prostate cells (normal, benign hypertrophic, and neoplastic) and seminal plasma [5, 6]. Original PSA Working Group [7] criteria for PSA progression remain unaltered from both PCWG2 and PCWG3 [8, 9]. Specifically, in 2016 PCWG3 recommended that treatment trial entry for metastatic CRPC be based on PSA doubling time (PSADT), and that the same standard imaging modalities (i.e. bone scan, CT, and/or magnetic resonance imaging- MRI) determining eligibility be applied for patient monitoring [1]. As an elusive protein, PSA cannot be utilized for imaging purposes.

Thus, conventional anatomical imaging, namely CT or MRI, is retained to determine invasion of lymph nodes within and beyond the true pelvis (short axis diameter ≥ 1.5 cm both abnormal and measurable, whereas 1.0 > nodes > 1.5 cm may be considered abnormal, but with clinical discretion) as well as visceral (lung, liver, adrenal, and CNS) disease. The recommended RECIST 1.1 criteria should be accompanied by documentation of the type of site-specific progression (growth of existing lesions versus development of new lesions). Overall, recording up to five lesions per site of spread is anticipated. Radionuclide bone scan using (technetium-99 m)technetium-diphosphonate compounds also remains the standard for identifying bone metastasis. Additional modalities assessing skeletal status such as PET imaging with sodium ^18^F-fluoride, ^18^F-fluorodeoxyglucose (FDG), choline or PSMA ligands, and bone marrow MRI can provide information about different intraindividual parameters, but the lack of standardized reporting on the presence of metastasis or the response to treatment, initially reserved these techniques as supplementary new biomarkers subject to independent endorsement.

### PSMA PET integration into medical societies’ guidelines

Rivetingly, the 750-amino acid type II transmembrane glycoprotein PSMA is overexpressed up to 1000 times in the cancer cells of almost all prostate adenocarcinomas [10–12]. The protein is involved in neuronal glutamate synthesis and encoded by the gene folate hydrolase 1 (FOLH1), thus also respectively called glutamate carboxy-peptidase II and FOLH1. Of note, insignificant concentrations of PSMA may be present in many organs. Apart from primary and metastatic prostate adenocarcinoma, high PSMA density may also appear in the neovasculature of various other malignancies [13–15]. Since 2009 the targeting of the catalytic site of PSMA in its large extracellular domain of PSMA with very small and highly specific γ- and β-radiolabelled urea-based anti-PSMA antibodies has offered a dominant nuclear medicine tool for prostate cancer (PCa) diagnostics and theragnostics, respectively [16–17].

Traditional single photon emission computed tomography (SPECT) or PET/CT with an intravenously injected PSMA-ligand are non-invasive diagnostic modalities indicated to visualise PSMA-positive lesions in men with PCa. Routine clinical use of either of the three approved PET tracers ([^18^F]fluorine-PSMA-1007, [^18^F]fluorine-DCFPyl, and [^68^Ga]gallium-PSMA-11) for medical practice, encompasses initial staging, restaging- recurrent (BCR) or persistent (BCP) prostate cancer, nmCRPC, and selection of patients to undergo PSMA-radioligand therapy [18]. No significant variations have been validated in the diagnostic yield of the three clinically approved tracers.

Image interpretation warrants optical evaluation of lesions which may or may not be accompanied by semi-quantitative analysis of the relative PSMA receptor density. Besides the parotid glands, reference organs for lesion assessment include the liver or spleen depending on the main elimination pathway of each radiopharmaceutical. Assessment standards such as the Prostate Cancer Molecular Imaging Standardized Evaluation (PROMISE) criteria have been developed to ensure structured and harmonized reporting [18–21]. The PROMISE criteria system (2018) assimilates pathological/clinical TNM staging and effectuates whole-body staging through the PSMA-expression score molecular imaging TNM (miTNM).

In nmCRPC, PSMA-positive lesions characteristically lack corresponding abnormalities on CT or bone scan. Optical PSMA-expression scoring of 0 (no uptake) to 3 (equal to or above the parotid gland) has been introduced by Eiber and colleagues for standardized reporting. Intermediate (2, i.e. equal to or above liver or spleen, depending on tracer elimination route, but lower than parotid) and high (3) score empirically designate PCa lesions and favour PSMA-directed radioligand therapy as opposed to uptake 0 and low (1, equal to or above blood pool and lower than liver or spleen). Notably for lymph nodes with high or equivocal PSMA expression but normal size (< 10 mm), prospective studies are still required to establish the best treatment option [22].

Expansion of PSMA PET usage is currently under consideration for guidance of prostate biopsy, as well as imaging and monitoring systemic treatment of metastatic PCa. Restaging men with nmCRPC, harnessing PSMA-ligand PET/CT has been the objective of several researchers [19, 23–25].

Increased PSMA expression in primary PCa has been associated with deficient DNA damage repair pathways [26] and other established poor prognostic parameters such as high tumor grade and pathological stage, aneuploidy, and biochemical recurrence (BCR) [27]. So, augmented PSMA expression occurs particularly in advanced metastatic and hormone-refractory PCa and is independently associated with adverse disease outcome [28]. Moreover, PSMA-overexpressing as compared with non-PMSA-overexpressing malignancies at the time of diagnosis have been associated with higher mean serum PSA levels [20, 27].

Nevertheless, depiction of PSMA upregulation was accomplished even at very low PSA levels (0.2 ng/dL) using PSMA-ligand PET/CT imaging in the BCR setting after radical prostatectomy (RP) due to high tracer binding and internalization [29, 30]. The high sensitivity and specificity of the method in the localization and stratification (local, locoregional, or distant) of BCR-linked cancerous tissue, urged all the associated medical societies to integrate PSMA-ligand PET/CT in their respective guidelines [31–34] (Table 2). The superior accuracy of the method over conventional imaging and ^**18**^F-choline [35] or ^**18**^F-fluciclovine [30] PET/CT, has been proved via prospective and retrospective studies with ^68^Ga/^18^F-PSMA-tracers. A detection rate exceeding 90% at PSA levels above 1 ng/mL [36] has been established. Higher PSMA PET/CT sensitivities have been coupled with higher PSA values, shorter PSADTs [37, 38], and higher initial Gleason scores [36]. Precise localization of disease extent by PSMA-ligand PET/CT may critically guide therapy and risk stratification in terms of individualized approach. In fact, staging high-risk PCa patients is strongly recommended to be conducted by PSMA PET/CT instead of bone scan and abdominopelvic CT, as concluded on the 2024 EANM Delphi consensus [39, 40] and in alignment with the 2021 Dutch consensus statement [41]. A positive PSMA PET permits targeted RT for oligometastatic disease and drives appropriate introduction of PSMA-directed radioligand treatment (RLT) or 2nd generation AR-oriented systemic therapies, such as abiraterone, enzalutamide, apalutamide, and darolutamide for metastatic CRPC (mCRPC). In case of local relapse, PSMA PET/CT readily identifies the disease site and guides salvage therapy, namely radiotherapy (RT) or surgery. A negative PSMA PET allows for a conservative “watch and wait” surveillance.

**Table 2:** Medical societies that integrated PSMA-ligand PET/CT in the respective guidelines for restaging prostate cancer in the setting of BCR [31–34]

Society	GY	Clinical scenario	SoR	Evidence Level
Joint **EANM** and **SNMMI**	2017	Especially recommended in patients with low PSA values between 0.2 and 10 ng/mL **to identify the site of recurrence and to potentially guide salvage therapy**	Strong	Retrospective
**EANM**- **EAU**- **ESTRO**- **ESUR**- **ISUP**- **SIOG**	2020	**After radical prostatectomy**, PSMA PET/CT if the PSA level is > 0.2 ng/mL and if the results will influence subsequent treatment decisions (EAU BCR risk groups)	Weak	2b
		**After radiotherapy**, PSMA PET/CT (if available) or fluciclovine PET/CT or choline PET/CT in patients fit for curative salvage treatment	Strong	2b
**ASCO**	2020	**Rising PSA after prostatectomy and negative conventional imaging** (either initial PSA undetectable with subsequent rise or PSA never nadirs to undetectable). For men for whom salvage radiotherapy is contemplated, nuclear imaging should be offered (PSMA imaging [where available], ^11^C-choline or ^18^F-fluciclovine PET/CT or PET/MRI, whole-body MRI and/or ^18^F-NaF PET/CT) as they have superior disease detection performance characteristics and may alter patient management. • For men with **BCR** after local therapy, who have negative conventional imaging, for whom salvage therapy is planned, nuclear medicine imaging is indicated to assess the presence of local or distant site and burden of disease, as this may alter treatment planning.	Strong	High
		• For men with nnCRPC, nuclear medicine imaging can be considered to assess for unrecognized metastatic disease, although the clinical impact on therapy is limited	Moderate (type of recommendation: consensus, benefits/harms ratio uncertain)	Weak
NCCN	2022	May be considered as an alternative to CT, MRI, and bone scans for initial staging of unfavourable intermediate-, high-, and very-high-risk disease, **the detection of biochemically recurrent disease**, and as workup for progression.	Strong	2a

BCR: biochemical recurrence, GY: Guideline Year, SoR: Strength of Recommendation, EANM: European Association of Nuclear Medicine, SNMMI: Society of Nuclear Medicine & Molecular Imaging, EAU: European Association of Urology, ESTRO: European Society for Radiotherapy & Oncology, ESUR: European Society for Urogenital Radiology, ISUP: International Society of Urological Pathology, SIOG: International Society of Geriatric Oncology, ASCO: American Society of Clinical Oncology, NCCN: National Comprehensive Cancer Network, nmCRPC: non-metastatic castration-resistant prostate cancer

Following androgen-signaling inhibition, PSMA expression has been found to upregulate in primary and especially in metastatic disease as well as the CRPC phenotype [42], a fact depicted by precociously enhanced lesion intensity on (^68^Ga)gallium-PSMA PET [43, 44]. The regulation of PSMA is multifaceted, with participation of androgen receptor (AR), direct effect on the PI3K/Akt growth pathways, and aberrant DNA damage response (DDR) pathways [14, 15, 26, 45]. PSMA overexpression in cells with deleterious DDR aberrations represents an adaptive cellular response and may be driven by the increased requirement of these cells for cellular metabolites such as folate and glutamate. The extracellular domain of PSMA potentially hydrolyzes glutamated folates released by dying tumor cells, creating folate which may be utilized by other PCa cells for proliferation [13]. The combination of PSMA overexpression and the antigen’s commitment in glutamate and folate metabolism may add to a survival advantage of tumor cells under cellular stress [46, 47].

## PSMA PET in nmCRPC

**Fig. 1:**
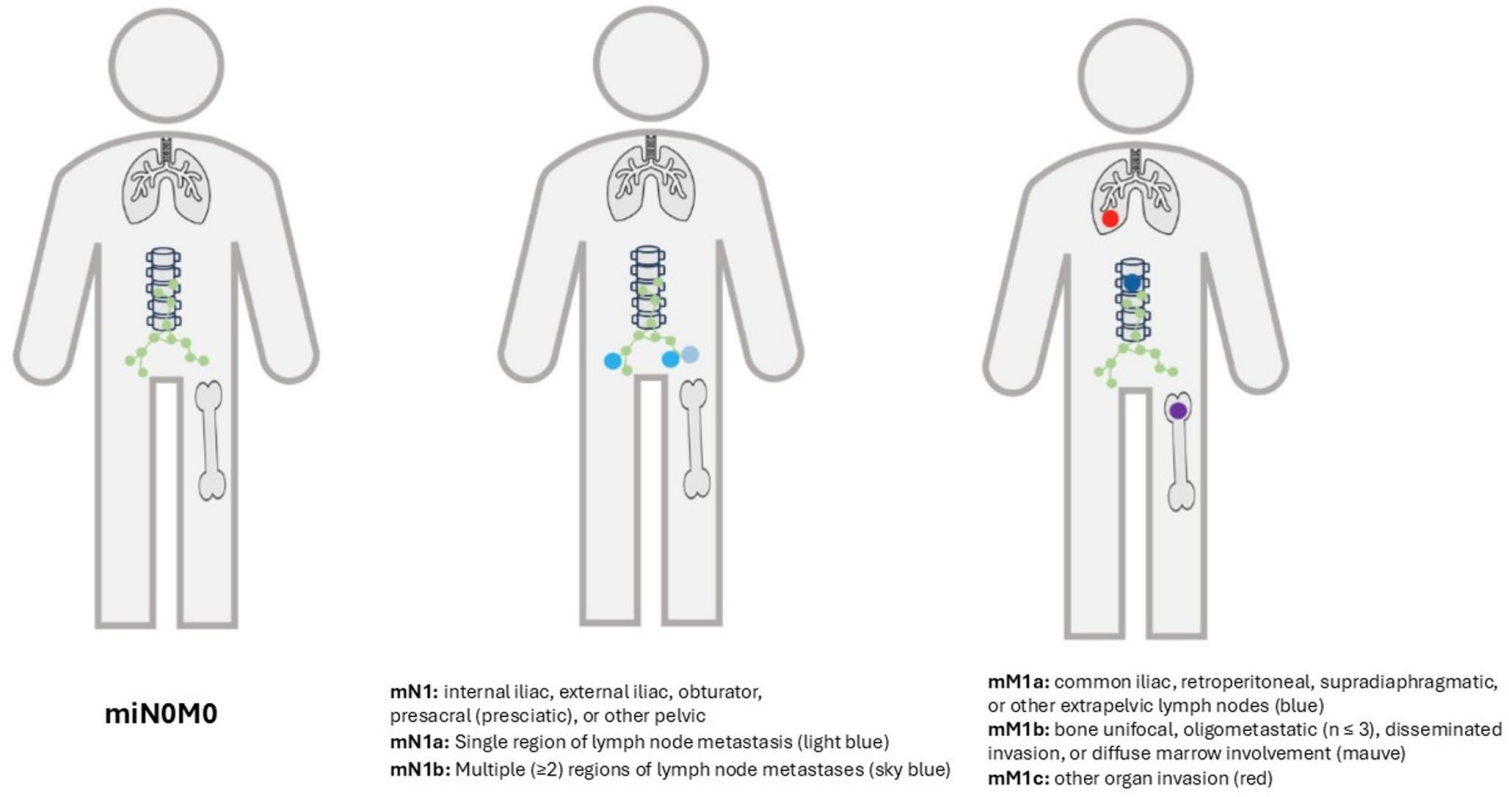
Illustration of miNM Classification for PSMA-Ligand PET/CT or PET/MRI [21]

Pertaining to the nmCRPC population, the aim is to inhibit the development of metastatic disease and prolong survival. The PCWG3 [1] first formally addressed a standardized diagnostic approach of nmCRPC to metastatic progression (Table 3) [48]. Notably, considering the uncertainty regarding the optimal method to confirm new metastatic bone disease at the time, the PCWG3 suggested the definition of metastasis-free survival (MFS) to be discussed with regulatory authorities if the MFS end point is required for a trial intended to support drug approval. The PROMISE staging regarding specifically lymph node (N) and bone or other organ invasion (M) through the PSMA-expression score molecular imaging (miNM), is vaguely illustrated in Fig. 1 [21].

**Table 3:** PCWG3 recommendations for nmCRPC to mCRPC progression [1, 47]

LNs size in short axis	LNs to be considered to have progressed: ≥1.0 cm and previously normal (< 1.0 cm) or abnormal in size that have grown by at least 5 mm from baseline or nadir	Abnormal and non-measurable, subject to clinical discretion: progression to 1.0 < LNs < 1.5 cm	abnormal and measurable LN: progression to ≥ 1.5 cm	For persistent lymphadenopathy, progression is defined per RECIST 1.1.
Visceral	The date of first metastasis: unequivocal visceral lesion by RECIST 1.1. No confirmatory scan is required unless the protocol uses modified criteria for immunotherapy			
Bone	Unambiguous development of new sites on bone scintigraphy Scanning interval in trials: 16 weeks (vs. 8 wk in mCRPC)		In the first post-treatment scan, possible flare of pre-existing subclinical metastatic lesion(s) or a true transition from a nmCRPC to CRPC; thus, treatment continued until two additional bone scan new lesions	

PCWG3: The Prostate Cancer Working Group 3

nmCRPC: non-metastatic Castration-Resistant Prostate Cancer

mCRPC: metastatic Castration-Resistant Prostate Cancer

Lymph Nodes: LNs

In nmCRPC, the PCa cells continue to progress despite ADT. Progression of the disease after ADT occurs in the bones in 95% of cases [49], implying that local factors in the bones facilitate the development of resistance to ADT [50]. The role of apoptosis as a possible mechanism for the therapeutic effect of ADT in hormone-sensitive PCa and also for ADT-augmented overexpression of PSMA in poorly differentiated and metastatic PCa, with subsequent development of hormone-independent PCa, insinuates a link under research in conjunction with bcl-2 and p53 [42]. Supposively, underlying micrometastases might be undetectable by conventional imaging [4].

The 2024 EANM Delphi consensus [39] strongly suggests PSMA PET at PSA below

1.5 ng/mL after RP/RT and in oligometastatic disease. In this subgroup of patients with low- volume mHSPC based on conventional imaging (≤ 3 bone metastases only in vertebral column or pelvis and absence of visceral metastases, criteria CHAARTED 1 and 2), there lies a strong EAU recommendation for ADT with local RT instead of ADT with androgen receptor pathway inhibitor (ARPI). About half of the patients with PSA range 0.2–0.49 ng/mL and more than one third of those with PSA below 0.2 present recurrence on (^68^Ga)gallium-PSMA PET scans [51]. In fact, a prospective study in the BCR context reported (^68^Ga)gallium-PSMA recurrence detection in 62.7% (197/314) of the patients [52]. Oligometastatic versus solitary lesions were detected in 43 patients (21.8%).

**Fig. 2:**
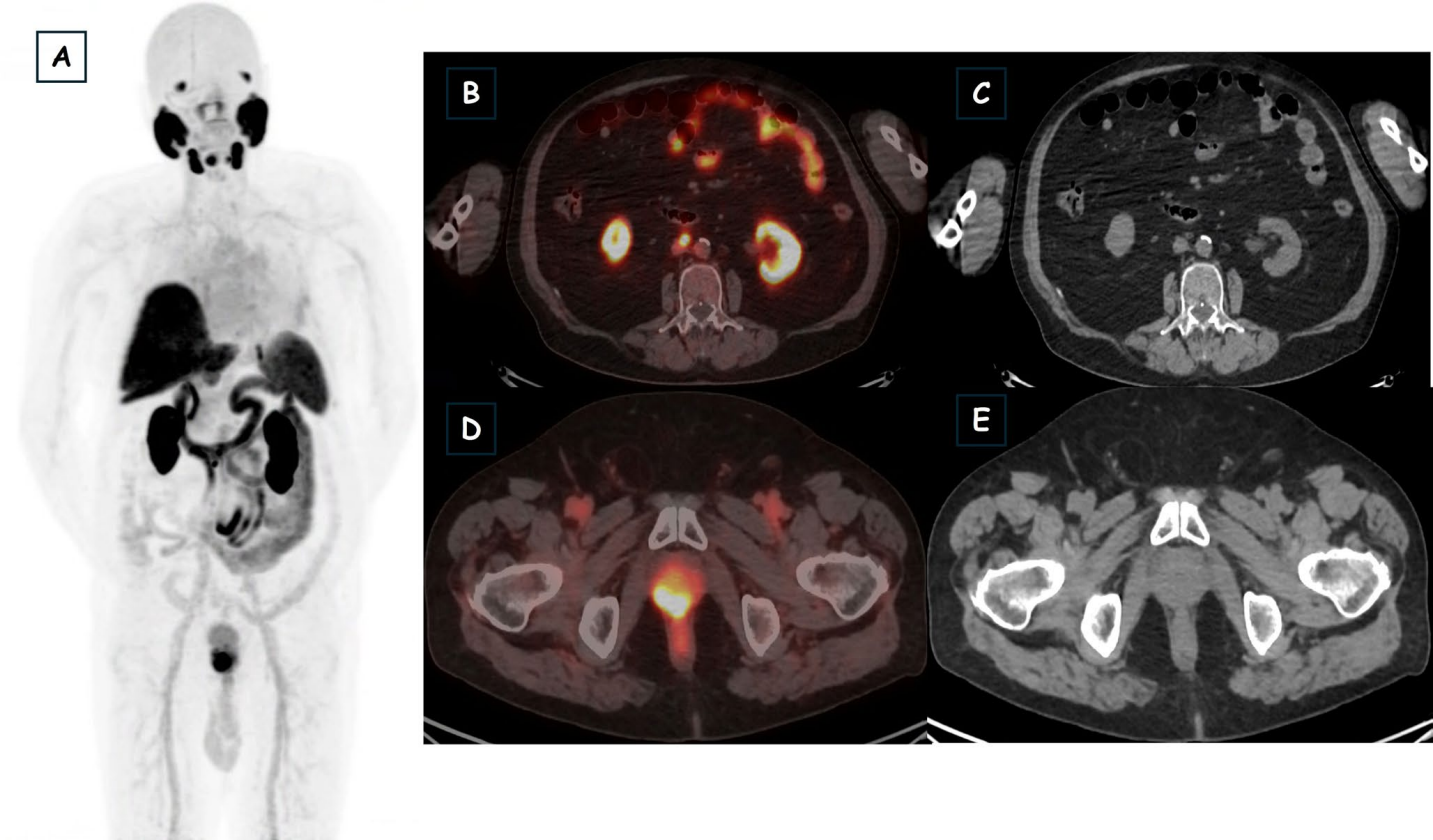
A 78-year-old man underwent ^18^F-PSMA-1007 PET/CT in our department due to biochemical recurrence. There were no suspicious lesions on abdominal CT despite rising PSA levels from 4.25 ng/mL to 5.57 ng/mL within a 3-month interval. The patient had been subjected to locoregional radiotherapy at the prostate bed and T9-T11 four years earlier and subsequently been treated with androgen deprivation therapy. The initial Gleason score was 8 (4 + 4). MIP (Panel A) and axial images (Panels B and D) show local relapse and a solitary extrapelvic (RP) lymph node metastasis, both with ^18^F-PSMA-1007 uptake higher than spleen but obscure on CT (Panels C and E). PROMISE code: miTr(3a)N0M1a, PSMA expression score lowest 2 (other lymph nodes not herein shown) and highest 3

An alternative PET tracer, ^**18**^F-fluorofluciclovine, should be used upon suspected post-RP local relapse. Specifically, the method is indicated at post-RP/RT PSA level above 4 ng/mL. Moreover, in the 2024 EANM consensus, a 70^th^ percentile agreement designated that the management of patients with nmCRPC (by conventional imaging) is likely to be modified by advanced imaging techniques (e.g. PSMA PET-CT/PET-MRI or whole-body MRI). Within this clinical scenario, ^18^F-PSMA-1007 PET/CT findings in a patient of ours are shown in Fig. 2; the modality identified local relapse and extrapelvic lymph node metastasis undetectable on CT, reorienting the therapeutic plan. Equally agreed, oligometastatic PCa should be defined as ≤ 5 metastases detected on these advanced imaging modalities.

Interestingly, in 2019 (^68^Ga)gallium-PSMA-11 or ^18^F-DCFPyL PET/CT has been found by Fendler et al., to identify any disease in almost all 200 nmCRPC patients with PSA > 2 ng/mL and risk features (high Gleason score ≥ 8 or short PSADT ≤ 10 months), i.e. locoregional only disease in 44% and M1 lesions in 55%, with a positive predictive value (PPV) of 96–97%, leading to considerable upstaging with high accuracy and reproducibility [19]. Notably, in this research, PSMA-PET has been determined to add important information to known risk factors since the PSMA PET M1 detection rate was not associated with PSADT or Gleason score levels but did correlate with pre-PET PSA, in accordance with a previous systematic review and meta-analysis.

In a 2020 smaller study by Fourquet and colleagues, the **(**^68^Ga)gallium-PSMA-11 PET/CT in nmCRPC patients, demonstrated 100% (20/20) positivity rate at PSA greater than 2 ng/ml, whereas only 70% (7/10) at PSA less than 2 ng/ml, with a sensitivity of 87% and specificity of 100% [23]. Furthermore, PSMA-11 PET/CT induced a change in disease management in 70% (60% at PSA less than 2 ng/ml), which was deemed adequate in 91% of patients. The authors suggested that PSMA-11 PET/CT should be contemplated on replacing bone scans. In addition, the results of the prospective phase 3 multicentre study CONDOR verified a correct localization rate (PPV plus anatomic lesion region coupling with a composite standard of truth) of 84.8%–87.0% for **(**^18^F)fluorine-DCFPyL-PET/CT in 208 patients with BCR (PSA ≥ 0.2 ng/mL after RP or ≥ 2 ng/mL above nadir after RT) but unrevealing standard imaging [53]. In descending order, the standard of truth entailed histopathology, subsequent corresponding imaging findings, or post-RT PSA response. An associated disease detection rate of 59%-66% and a 63.9% impact of **(**^18^F)fluorine-DCFPyL-PET/CT in management of evaluable patients were determined.

In a recent prospective co-evaluation of high-risk nmCRPC patients with an early PSA progression under castration after RP, applying both (^68^Ga)Gallium-PSMA and **(**^18^F)fluorine-FDG PET/CT, Wang and colleagues observed a high prevalence (73%) of N- positive/M-positive disease and a significant proportion of PSMA-negative coupled with FDG-positive disease [24]. The median PSA at imaging was 0.57 ng/mL. Overall, 114 lesions were detected in 29 of the 37 patients, 71.1% PSMA-positive/FDG-negative and the rest vice versa. A statistically significant correlation was established between a short PSA doubling time and PSMA-positive/FDG-negative disease, whereas a high Gleason grade was associated with PSMA-negative/FDG-positive disease. Enrolment for treatment of oligometastatic disease was eligible in 51% of the patients, including 19.6% PSMA- negative/FDG-positive lesions.

Interestingly, the PCa cells which undergo dedifferentiation and attain the neuroendocrine phenotype, have been ascertained with PSMA-negative but FDG-positive imaging [54]. The presence of PSMA-negative/FDG-positive PCa lesions implies aggressive biology. This dual tracer approach aids in improved risk stratification and treatment planning in the advanced or recurrence context. Some authors advocate that dual-tracer PET/CT may be valuable even in preoperative risk stratification to differentiate patients with aggressive disease who might benefit from neoadjuvant or intensified adjuvant treatment [54].

Considering that, unfortunately, no imaging modality is “panacea”, a false-negative PSMA PET/CT scan result besides neuroendocrine dedifferentiation of PCa (overall 5% false-negative PSMA PET), may be attributed to physiological liver uptake obscuring hepatic metastasis, late-stage downregulation of PSMA expression, or PET spatial resolution. Standard clinical PET achieves a resolution of 4 mm, hindering recognition of smaller nodal or organ disease. The drawbacks of false negatives, in terms of radical treatment of incurable patients, are obviously not limited to PSMA PET and accordingly apply to all the existing imaging modalities. In a prospective study investigating ^68^Ga-PSMA-11 PET accuracy in the recurrent setting, false negatives were proven by biopsy and/or surgery in merely 8/635 patients [37]. Of note, 4 cases with faint focal uptake, 3 with CT/MRI lesions (mean size, 0.9 cm) and 1 with clinical suspicion were encountered in the prostate bed (SUVmax 7.2), left seminal vesicle (SUVmax 5.5), retroperitoneal (SUVmax 4.5)/mesorectal lymph node, or lung lesions.

False-positive findings are more commonly encountered and include the endothelial angiogenesis of non-prostate hematologic (such as Hodgkin lymphoma) or solid tumors (e.g. primary lung cancer, differentiated thyroid cancer, hepatic, pancreatic or urothelial carcinoma), benign processes (meningioma, hyperplastic thyroid nodules, pulmonary nodules/infiltrates, adrenal adenoma, fractures, osteophytes, enchondroma, fibrous dysplasia, Paget’s disease, haemangioma, atrial lipoma, lymph nodes axillary, mediastinal, and cervical), non-specific iliac lymph node, thyroid diffuse or rib uptake (i.e. lacking anatomical counterpart), sympathetic ganglia, and AR inhibition [55, 56]. Evidently, correlation of abnormal PSMA PET uptake with anamnesis and any previous or concurrent CT/MRI findings is mandatory.

The hitherto PSMA PET proven stage migration of nmCRPC patients, infers important considerations concerning treatment and prognosis. In asymptomatic nmCRPC patients, who are not eligible for curative therapy, the application of systemic ADT remains the standard of care for patients with PSA doubling time > 10 months [57]. Furthermore, in SPARTAN, PROSPER, and ARAMIS studies, the addition of androgen receptor inhibitors, such as apalutamide, enzalutamide, and darolutamide, to ADT has been shown to improve MFS in nmCRPC with PSA doubling time < 10months [57–62]. Based on PSMA PET restaging, especially in high-risk nmCRPC, the combinations of (^177^Lutetium)Lu-PSMA radioligand therapy (RLT) with novel hormonal agents in metastatic advanced PCa, play at present mainly a role outside of clinical trials [61–65]. According to the recently published results of the phase II randomized multicentre trial, EnzaP, the median PSA PFS was 13.0 months (95% CI: 11.0–17.0) in the enzalutamide plus RLT group of 83 patients and 7.8 months for 79 patients assigned to enzalutamide alone [66].

On the other hand, PSMA PET-guided local salvage strategies, i.e. stereotactic body RT (SBRT), may add synergistic advantage to systemic androgen-signaling inhibition in this patient population [19]. The recruiting PEACE 8, a phase III randomised multicentre trial, evaluates the benefit of adding SBRT to darolutamide for treating patients with oligometastatic CRPC, with metastasis visible only on PSMA PET and not on CT-scan or bone scintigraphy [67]. The mitogen activated protein kinase pathway- MAPK or MAPK/extracellular signaling-regulated kinase (MAPK/ERK or RAS-RAF-MEK-ER) is often dysregulated in PCa and can thus affect cell fate, including survival, apoptosis, migration and invasion, with resultant metastatic spread of PCa cells. In advanced disease, metastases (especially liver) may lose PSMA expression, possibly directed by mutations.

Evidence suggests that the Her2/Raf-1/MAPK/AP-1 axis may promote the development of CRPC, leading to early relapse, and reduced disease-specific survival [68]. Also, activation of MAPK signaling through the atypical chemokine receptor CXCR7 can lead to resistance to enzalutamide, highlighting the therapeutic potential of MAPK/ERK inhibitors in CRPC [69]. Furthermore, p38 MAPK regulates the Wnt inhibitor Dickkopf-1 (DKK-1) in osteotropic prostate cancer by inhibiting osteoblastogenesis and may present a potential target in osteolytic metastases [70]. PSMA can directly activate the Phosphoinositide 3-kinase/Protein Kinase B (PI3K/Akt) signaling pathway through glutamate release and subsequent binding on glutamate receptors, specifically metabotropic glutamate receptor mGluR1. In a reciprocal feedback loop, inhibiting AR signaling can activate PI3K/Akt and vice versa, a bidirectional key mechanism promoting tumor cell survival and resistance to therapy in PCa.

The extraordinary malleability of tumor cells adjusting and evading treatment in nmCRPC, albeit complex and multifactorial, seems inherently linked with the gradual development of the phenotypic subpopulation of PSMA-expressing PCa cells. The obviously multifaceted cellular processes and crosstalk, including the influence of mutations, involved in PSMA overexpression are under investigation to elucidate pharmacological strategies that can manipulate PSMA expression aiming at optimal drug combinations for PSMA-targeting therapeutics. Such approaches have been suggested and include generating PCa genomic instability or replication stress [26].

Blocking androgen signaling with enzalutamide or abiraterone, has been shown to increase PSMA expression but is considered unsuitable due to associated significant side effects in patients and high costs for the health care system [71]. At the same time, targeting the mevalonate pathway via lovastatin in LNCaP and VCaP can induce PSMA expression and elevate HOXB13 levels as well as inhibit AR and mTOR in prostate cancer cell, suggesting that there are other critical regulators of PSMA besides AR. In both prostate cancer cell lines and mouse models, treatment with dutasteride, used for lower urinary tract symptoms caused by benign prostatic hyperplasia, has been found to enhance PSMA expression in a dose- and time-dependent pattern and increase HOXB13 without an inhibitory effect on AR. These findings corroborated HOXB13 as a positive regulator of PSMA expression.

Another conceivable option might focus on disruption of the signaling growth pathway Ras/PI3K/Phosphatase and tensin homolog/Akt/mammalian target of rapamycin (or Ras/PI3K/PTEN/Akt/mTOR) with protein kinase inhibitors (PKIs) targeting different levels along this pathway, which might render PCa cells redirected, in terms of a negative feedback cascade upregulating the PSMA for PSMA-ligand PET detection. For example, ipatasertib, a compound targeting the serine/threonine kinase Akt, has yielded excellent preliminary therapeutic results coupled with a favorable safety profile in patients who have lost PTEN [72]. At the same time, ipatasertib upregulates the MAPK/ERK pathway. PSMA can counteract this effect via interaction with RACK1 protein, which prevents the formation of a complex that activates the MAPK/ERK pathway, hence shifting the stimulation towards PI3K/Akt; this switch may represent resistance to treatment. The underlying mechanisms may also engage preexisting or de novo mutations of the resistant cancer cells. Angus and colleagues emphasised the epigenetic changes underlying resistant phenotypes and described phenotypic switching as an adaptive response to kinase inhibition [73]. Since HOXB13 promotes the expression of Akt, Akt kinase inhibitors can block this downstream increased cell proliferation and resistance to apoptosis but do not affect the HOXB13-mediated PSMA upregulation. Finally, a cooperative effect of HOXB13 and AR in regulating PSMA has been verified in LNCaP cells [71].

In clinical praxis, integration of PSMA-ligand PET/CT has resulted in upstaging of a significant proportion of patients, but the corresponding suitable management (systemic therapies or aggressive local and metastasis-directed therapies) and associated prognosis remain ambiguous. Furthermore, the crossover from a group with a better prognosis to the group with worse prognosis may produce misunderstanding of survival data. This familiar and expected epidemiological paradox was first described by Dr. Feinstein in 1985 adhering to the clinical integration of the CT scan in lung cancer patients (NEJM) and adopting the term “Will Rogers phenomenon” after the inventive comedian [74, 75]. Quoting Will Rogers, «when the Okies left Oklahoma and moved to California, they raised the average intelligence level in both states» [76]. Nevertheless, comprehension and interpretation of medical data is an objective, not statistically confounded, process. The PSMA-ligand-related Will Rogers phenomenon statistically reflects averages within groups and ignores the better evincement of disease reality and the respective guided management by this novel conceptual modality.

*In conclusion*, the introduction of PSMA PET/CT into the routine clinical practice for prostate cancer patients is likely to eliminate the stage of nmCRPC. This could usher in amending the precision management of CRPC patients with a potential benefit in overall survival. Intriguingly, pathways towards the phenotypic modification to reveal adequate PSMA density even in the small fraction of nmCRPC with micrometastases are partly being explored. More prospective studies are warranted to confirm this review’s challenging issue.
